# Potential acetylcholine-based communication in honeybee haemocytes and its modulation by a neonicotinoid insecticide

**DOI:** 10.7717/peerj.17978

**Published:** 2024-09-13

**Authors:** Tobias Pamminger, Kate Basley, Dave Goulson, William O. H. Hughes

**Affiliations:** 1 School of Life Sciences, University of Sussex, Brighton, UK; 2 Bayer AG, Monheim am Rhein, Germany

**Keywords:** Haemocytes, Pesticide, Innate immune system, Immune regulation, Clothianidin, Neonicotinoid, Bee health, Immunosuppression

## Abstract

There is growing concern that some managed and wild insect pollinator populations are in decline, potentially threatening biodiversity and sustainable food production on a global scale. In recent years, there has been increasing evidence that sub-lethal exposure to neurotoxic, neonicotinoid pesticides can negatively affect pollinator immunocompetence and could amplify the effects of diseases, likely contributing to pollinator declines. However, a direct pathway connecting neonicotinoids and immune functions remains elusive. In this study we show that haemocytes and non-neural tissues of the honeybee *Apis mellifera* express the building blocks of the nicotinic acetylcholine receptors that are the target of neonicotinoids. In addition, we demonstrate that the haemocytes, which form the cellular arm of the innate immune system, actively express choline acetyltransferase, a key enzyme necessary to synthesize acetylcholine. In a last step, we show that the expression of this key enzyme is affected by field-realistic doses of clothianidin, a widely used neonicotinoid. These results support a potential mechanistic framework to explain the effects of sub-lethal doses of neonicotinoids on the immune function of pollinators.

## Introduction

Pollinating insects such as bumblebees are of major ecological and economic importance, but many of their populations are in decline (Potts et al., 2010; Vanbergen et al., 2013). Threats include natural diseases and emerging diseases related to the globalized pollinator-trade that have negative effects on both managed and wild pollinator populations (Fürst et al., 2014; Goulson & Hughes, 2015; McMahon et al., 2015; Wilfert et al., 2016). While healthy pollinator communities are sometimes able to cope with such diseases, additional stressors can compromise pollinator immunity, potentially resulting in lethal epidemics (Botías et al., 2021; Goulson et al., 2015). One factor that is now known to impact pollinator immunity is their exposure to sub-lethal doses of pesticides. Many studies have now demonstrated that exposure to neurotoxic neonicotinoids in particular, can significantly impair multiple components of the cellular and humoral immune response, with consequent effects on parasite replication (Annoscia et al., 2020; Brandt et al., 2016; Di Prisco et al., 2013; López et al., 2017; Malladi et al., 2023; Orčić et al., 2022). While the detrimental effects of neurotoxic pesticides on pollinator behaviour and navigation are intuitive (Fischer et al., 2014; Henry et al., 2012; Jin et al., 2015; Stanley et al., 2016), the strong immunosuppressive effects of these neurotoxic pesticides are difficult to mechanistically explain (Sanchez-Bayo et al., 2016). The close ontogenetic connection between haemocytes and the nervous system has been proposed as a possible explanation (Pamminger et al., 2018), with haemocytes having been shown to express receptors to biogenic amine neurotransmitters for example (Huang et al., 2012; Qi et al., 2016), but this mechanism remains to be investigated.

In vertebrates it is well established that the immune system has a close regulatory connection with the nervous system (Sternberg, 2006). In particular, the ancient cholinergic signalling system based on acetylcholine (*ACh*) has been demonstrated to perform a pivotal role in maintaining homeostasis of the immune system (Kawashima et al., 2012; Sternberg, 2001). Evidence for a functionally similar *ACh*-based immune regulatory network has more recently emerged in several bivalve mollusc, crustacean and insect species (Chen et al., 2016; Giordani et al., 2023; Shi et al., 2014, 2012; Zhang et al., 2021). In particular, haemocytes, the cellular arm of the invertebrate immune system, have been demonstrated in oysters to not only express subunits of the muscarinic (*mAChR*) and nicotinic acetylcholine receptors (*nAChR*), but also to directly respond to the presence of *ACh* (Chen et al., 2015; Liu et al., 2016b). In insects, haemocytes have also been found to express and have receptors for *nAChR* and *ACh*, with knockdown of *Ach* synthesis in *Drosophila* haemocytes reducing expression of a gene for antimicrobial peptide production (Giordani et al., 2023; Xu et al., 2017). Since neonicotinoids, and other insecticides, target *nAChR* receptors with high affinity (Christen & Fent, 2017; Tomizawa & Casida, 2003), the presence of a neural-independent, *ACh*-based communication system in the innate immune system of pollinators could provide a direct mechanistic link for immunosuppression by neonicotinoids and other insecticides (Pamminger et al., 2018).

In this study, we investigate if non-neural immune-relevant tissues (fatbody, midgut and haemocytes) of the honeybee *Apis mellifera*: 1) express *nAChR* subunits and choline acetyltransferase (ChAT), a key enzyme to synthesize *ACh*, and 2) if such a system can respond to a field-realistic dose of neonicotinoid that bees could encounter under natural conditions.

## Methods

Portions of this text were previously published as part of a preprint (https://doi.org/10.1101/105700).

### Bee collection

Foraging *Apis mellifera* worker were collected between July and September 2016 from a single colony on the campus of the University of Sussex, Brighton, UK (50°52′02.8″N 0°05′09.6″W). We used foragers because they have mature immune systems and are the bees most directly exposed to neonicotinoids on flowers. In all cases bees were collected between 09:00 and 11:00 in order to minimize gene expression variation caused by circadian rhythms. They were placed in 50 mL Falcon tubes with three bees per tube. The tubes contained a moist cotton ball as a water source and to regulate relative humidity within the tube.

### Experiment 1: tissue-specific expression of *nAChR* subunits and *ChAT*

The bees collected for Experiment 1 (tissue expression levels) were directly put on ice to cold anaesthetise them. After cold immobilization (~10 min) the bees were decapitated using a sterile razor blade, dissected under RNA Later (Thermo Fisher Scientific, Waltham, MA, USA) using a sterile dissection kit and either whole brain (*N* = 5), fatbody (*N* = 7) or midgut (*N* = 7) was extracted (one type of tissue per bee). For haemolymph extraction, the thorax and abdomen of the bees were carefully punctured after decapitation using a sterile dissection needle and haemolymph was collected using a sterile graded glass capillary. The haemolymph of two bees was pooled (total 16 bees; *N* = 8) and haemocytes were collected following standard protocol (Negri et al., 2014). All tissues were homogenized in Trizol (ABI, New York, NY, USA) using a sterile pestle and total RNA was extracted following the manufacturer’s instructions. The concentration and purity of RNA was determined on a Nanodrop 2000^®^.

### Experiment 2: clothianidin exposure

To test whether neonicotinoid exposure affects *ChAT* expression, sixty-two foraging *A. mellifera* workers were collected and randomly assigned to either treatment (*N* = 30) or control (*N* = 32). After collection, the bees were placed in their Falcon tubes in a dark incubator at 33 °C and 80% relative humidity. The tubes had a hole drilled in one end through which the bees were provided with 60% sucrose solution *via* 10 mL syringe feeders. The bees were kept at these conditions for 20 h for acclimatisation before the start of the experiment. Following the 20 h acclimatisation period, the feeders were removed. A total of 4 h later the treatment group was provided with new feeders containing 60% sucrose solution spiked with 5 ppb clothianidin (using molecular grade acetone as solvent), while the control received sucrose solution with the same concentration of acetone only. The clothianidin concentration was chosen as a field-realistic exposure level, within the range of residue levels reported for treated crops (Botias et al., 2015; Sanchez-Bayo & Goka, 2014), and resulted in bees ingesting a dose that was approximately an order of magnitude less than the oral LD_50_ of 3.8 ng/bee (Bartling, Vilcinskas & Lee, 2019; European Commission, 2005). All feeders were weighed before and after the experiment to the closest 0.001 g using a Kern PFB 300-3 scale to measure the dose (ng) of neonicotinoids that the bees had consumed. All bees had access to the feeders for 24 h after which haemolymph was collected from all surviving individuals, and samples of two bees were pooled (resulting in *N* = 6 treatment, *N* = 7 control) following the procedure of Experiment 1.

### Gene expression analysis

We quantified expression levels for the nine *α* (*1-9*) and two *β* (*1-2*) subunits of *nAChR* (Jones et al., 2006), and *ChAT*. We compared these to the reference gene *rp49* (Lourenço et al., 2008). We used 100 ng of total RNA for reverse transcription using the Phusion RT-PCR kit (Thermo Fisher Scientific, Waltham, MA, USA). The purity of the RNA samples was checked using a Nanodrop (Thermo Fisher Scientific, Waltham, MA, USA) based on absorbance curve and the 260/280 and 260/230 ratios. Primers for all the target genes were designed using Primer3 (Untergasser et al., 2012) and published sequences available from GenBank (see Table S3 for details). Primer efficiencies were measured using a dilution series of *Apis mellifera* brain cDNA (pooled subsamples of five individuals) covering three orders of magnitude including the cDNA concentration used in the reaction. Primer efficiencies were found to be above 91% for all primer pairs. Reaction specificity was confirmed by melting curve analysis. All analyses were performed on a StepOnePlus^™^ Real-Time PCR system (Applied Biosystems, Waltham, MA, USA) using SYBR green assays and were analysed using the StepOne software.

Gene expression analysis of the *nAChR* subunits *α1-9, β1-2, ChAT* and *rp49* were performed in 10 μL reactions using GoTaq® qPCR Master Mix (Promega, Madison, WI, USA) and 0.5 μM of each primer (Sigma-Aldrich, St. Louis, MO, USA) on the StepOnePlus^™^ Real-Time PCR System. Samples of cDNA corresponding to 2 ng total RNA in 2 μL volumes were added and each sample analysed in three technical replicates. Each plate contained one negative control reaction for each primer pair using pooled and 1:10 diluted RNA extracts from five randomly chosen individuals in order to control for gDNA contamination. The following program was used for amplification: 95 °C for 2 min, followed by 40 cycles of 30 s of 95 °C denaturation, 30 s annealing at 59 °C and 30 s extension at 72 °C following by a melting curve to ensure PCR specificity. The data used for the analysis were the target gene expression normalized to the *rp49* reference gene expression using averages of the technical replicates and the 2^−ΔΔ*CT*^ method (Rao et al., 2013). Note that although the *rp49* reference gene shows good expression stability across honeybee tissues (Lourenço et al., 2008), the use of only a single reference gene means that comparisons in absolute expression levels between tissues should be interpreted cautiously.

### Data analysis

To compare the *nAChR* subunit expression patterns we used the programme PRIMER 6, version 6.1.13, + add-in, version 1.0.3 (PRIMER-E Ltd) to perform permutational multivariate analysis of variance (PERMANOVA) with the normalized relative expression of all 11 subunits as the response and tissue as the predictor variable. PERMANOVA is free of assumptions about variable distributions (Anderson, 2017). All tests were carried out using 9,999 permutations on a resemblance matrix using Chad distance estimates and the robustness of the results were tested using the Euclidian distance as an alternative estimate. We performed a SIMPER analysis to compare the expression of individual *nAChR* subunits according to tissue identity and tissue differentiation. All other tests were performed in R 3.2.4 (R Development Core Team, 2015). Survival was analysed as the proportion of bees that died over the duration of the experiment using a GLM with binomial data distribution. The other results of Experiment 2 were analysed using non-parametric statistics (Kruskal-Wallis and Wilcoxon tests) and Bonferroni corrections in cases of multiple testing. The MDS plot was generated in PRIMER 6; all other graphs were done in R using the sciplot package (Morales, 2011).

## Results

**Figure 1:**
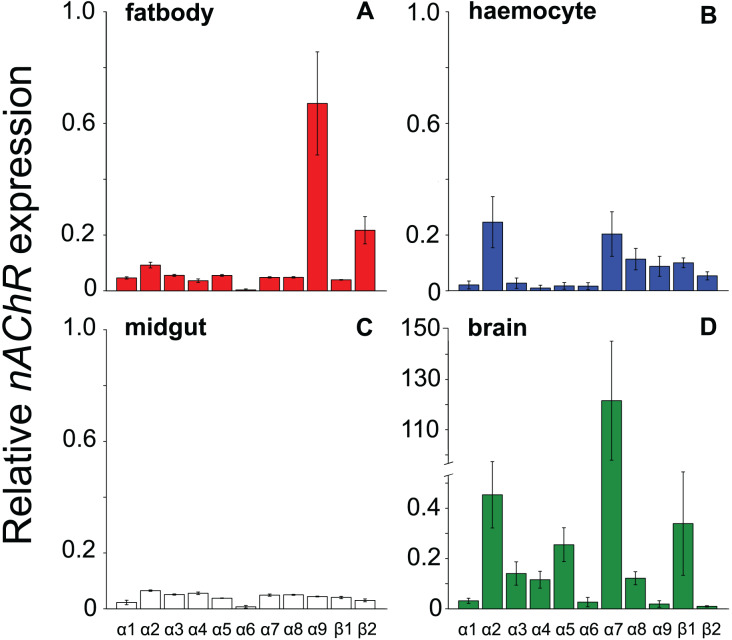
Mean ± s.e. relative expression of the *nAChR* subunits *α1-9* and *β1-2* in honeybees. Expression values were normalized against *rp49*. Data are for fatbody (A in red, *N* = 7); haemocytes (B in blue, *N* = 8); midgut (C in white, *N* = 7); brain (D in green, *N* = 5; note the much higher expression of *a7* indicated by the break in the y-axis).

**Figure 2:**
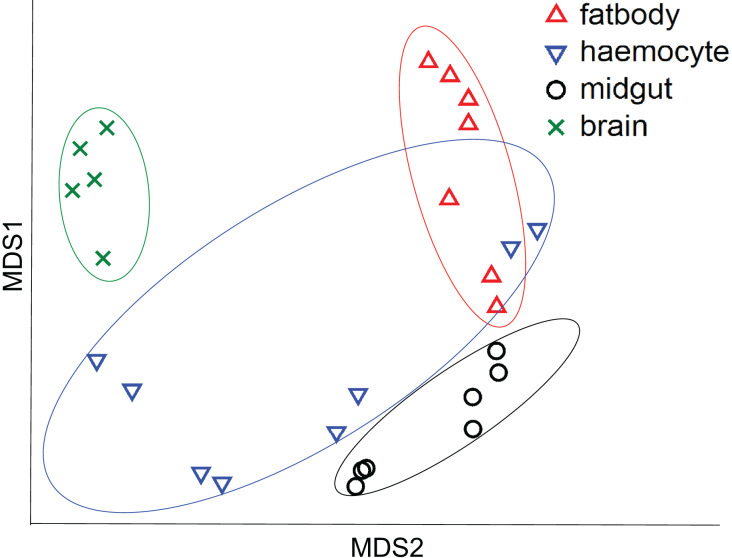
Multidimensional scaling (MDS) plot of *nAChR* expression in honeybees. Plot is based on Chad distance. Data are for brain (green crosses, *N* = 5), haemocytes (blue inverted triangles, *N* = 8), fatbody (red triangles, *N* = 7) and midgut (black circles, *N* = 7).

**Figure 3:**
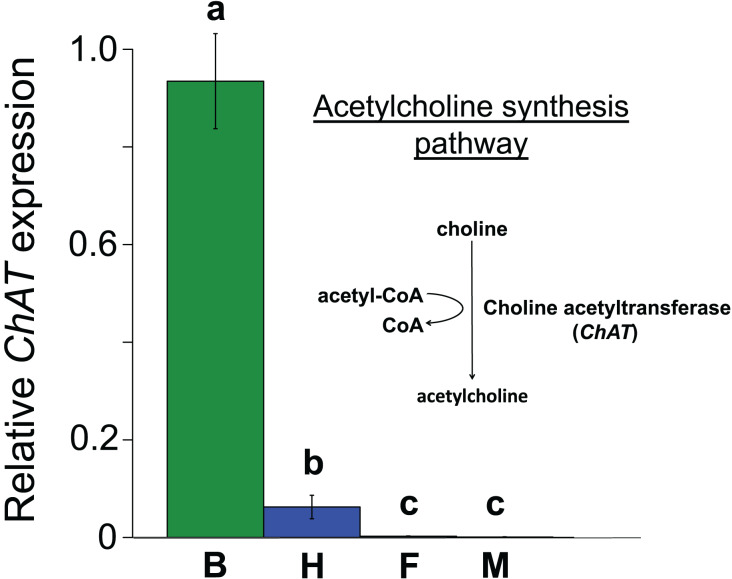
Mean ± s.e. relative expression of the *A. mellifera* choline transferase gene (*ChAT*) in honeybees. Data are for brain (green, B, *N* = 5); haemocytes (blue, H, *N* = 8); fatbody (red, F, *N* = 7); midgut (white, M, *N* = 7). The inset shows the role of *ChAT* in acetylcholine synthesis. Only brain and haemocyte cells exhibit robust *ChAT* expression. Different letters above columns indicate significant expression differences between cell types.

All the investigated tissues (fat body, haemocyte, midgut, brain) expressed *nAChR*, with the relative expression pattern of the different subunits differing between tissues (Pseudo-F_3,23_ = 7.76, *P* < 0.001; Figs. 1A–1D). The expression patterns of the subunits differed significantly between all four tissues in pairwise comparisons (t > 1.92 and *P* < 0.006 in all cases; Table S1). In the brain, *α7* was very highly expressed compared to the other subunits, with *α2* and, to a lesser extent, *α5* and *β1* being moderately expressed relative to the remaining subunits (Fig. 1D). Expression in the haemocytes was also highest for *α2* and *α7*, with *α8*, *α9*, *β1* and *β2* being higher than the remaining subunits (Fig 1B). Expression in the fatbody was dominated by *α9* and *β2* (Fig. 1A), while expression in the midgut was relatively low and similar for most of the subunits (Fig. 1C). Brain, midgut and, to a lesser extent, fatbody samples formed distinct clusters in multidimensional scaling plot, with the haemocyte samples occupying a larger area that overlapped partially with fatbody (Fig. 2). The SIMPER analysis indicates that the largest differences between tissues, in terms of the expression pattern of the different subunits, were between the brain and the other tissues, with the very high expression of *α7* in the brain relative to other subunits being a consistent cause of this (Table S2). We also found evidence of expression of *ACh*, measured as choline acetyltransferase (*ChAT*) expression, with the expression being very high in the brain samples, lower in the haemocytes and very low in the midgut and fatbody (Fig. 3). The expression levels of *ChAT* differed significantly between the tissues (χ^2^ = 21.96, *P* < 0.001; though note this could also relate to differences between tissues in expression of the reference gene).

**Figure 4:**
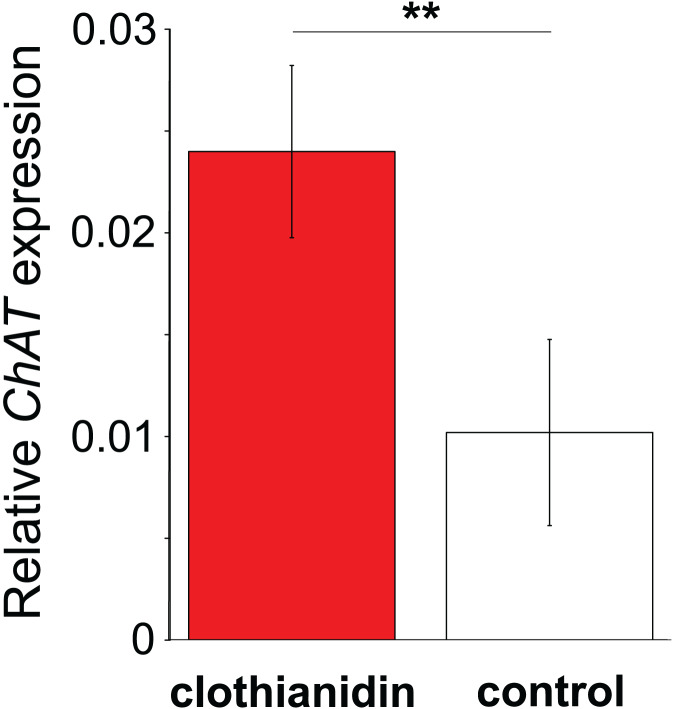
The effect of clothianidin on choline transferase (ChAT) expression in honeybee haemocytes. Graph shows the mean ± SE relative expression of *ChAT* normalised against the *rp49* reference gene. Bees were either treated with the neonicotinoid clothianidin (red; *N* = 6) or control (white; *N* = 7). Asterisks above the columns indicate that expression in clothianidin and control bees differed significantly.

In Experiment 2, Treatment and Control bees consumed similar amounts of sucrose solution (mean ± s.e. 0.064 ± 0.001 and 0.06 ± 0.001 mL per bee respectively, W = 114, *P* = 0.95). This equated to a consumption of 0.3 ± 0.008 ng of clothianidin by the Treatment bees. Treatment and Control bees did not differ in survival (z = −0.26, *P* = 0.79), with mortality being relatively high in both cases (16/32 bees and 16/30 bees, respectively). Importantly, we found that *ChAT* expression was significantly increased in the haemocytes of bees exposed to clothianidin (W = 38, *P* = 0.014), with the expression levels in bees treated with clothianidin being almost 2.5 times higher than in Control bees, Fig. 4).

## Discussion

In this study we demonstrate the widespread expression of *nAChR* subunits in non-neural and immune-relevant tissues in the honeybee *A. mellifera*. In addition, we show that haemocytes in *A. mellifera* express the key enzyme to synthesize *ACh*, which suggests that in principal all components for an *ACh*-based communication (receptor and signalling molecule) are expressed. Lastly, we experimentally establish that sub-lethal, field realistic doses of the neonicotinoid clothianidin can influence the expression pattern of the ChAT communication system *in vivo*.

Our results are in line with recent findings, which suggest the presence of an non-neural and immune-related *ACh-*based communication in a range of invertebrates (Chen et al., 2015; Giordani et al., 2023; Liu et al., 2016b; Shi et al., 2014; Zhang et al., 2021). Similar to our findings, different combinations of *nAChR* subunits have been found to be expressed in a wide range of non-neural tissues in a lepidopteran insect (Xu et al., 2017). In both our results and those of Xu et al. (2017), *α7* was the dominant subunit in the brain, *α9* and *β2* were the most highly expressed subunits in the fatbody, and all subunits had very low expression in the midgut. We found *α2* and *α7* to be the most highly expressed subunits in honeybee haemocytes, whereas Xu et al. (2017) found *α3* to be the most highly expressed subunit in the lepidopteran haemocytes, with *α2*, α7 and the other subunits having similarly low expression. The expression of these sub-units by itself does not automatically indicate the presence of functional receptors (Aztiria, Sogayar & Barrantes, 2000). However, the fact that haemocytes can respond to the presence of *ACh* in molluscs suggests that, at least in some species, functional receptors are most likely present (Liu et al., 2016b; Shi et al., 2014). In addition, haemocytes have been shown to synthesize acetylcholine-degrading enzymes (acetylcholinesterase) in scallops, likely terminating *ACh*-based haemocyte excitation following pathogen exposure (Shi et al., 2012), and expression of the *nAchR* subunit *α7* in haemocytes has been shown to be necessary for production of an important antimicrobial peptide in *Drosophila* fruit flies (Giordani et al., 2023). Our results are in keeping with these findings and indicate additionally that haemocytes in principal may express the enzymatic machinery to actively synthesize *ACh* themselves. Taken together these lines of evidence suggest that invertebrate innate immune systems may possess all the essential components for sending, receiving and terminating *ACh* based signals. It is consequently possible that, similarly to their vertebrate counterparts (Kawashima et al., 2012), the invertebrate innate immune system utilizes *ACh*-based communication.

Subunits of *nAChR* subunits were also expressed by secondary immune-relevant tissues, the fatbody and the midgut (Xing et al., 2021; Zhu et al., 2022). This was similarly the case in a lepidopteran stem borer (Xu et al., 2017), with expression of all subunits being similarly low in the midgut, while *α9* and *β2* were relatively highly expressed compared to other subunits in the fatbody. It would be interesting to investigate whether haemocytes could utilize *ACh*-based signals to convey information to the fatbody and midgut, thereby coordinating the systemic immune response during infections.

In addition to establishing that honeybee haemocytes express *nAChR* subunits and *ChAT*, we found that exposure to the neonicotinoid clothianidin affected *ChAT* expression in the haemocytes of honeybees. We found this effect at a dose of 0.3 ng/bee, which is approximately an order of magnitude less than the LD_50_ of 3.8 ng/bee (Bartling, Vilcinskas & Lee, 2019; European Commission, 2005; Lewis et al., 2016). It has been experimentally shown that clothianidin at 10 ppb negatively affects the encapsulation, melanisation and antimicrobial immune properties of haemolymph in honeybees, with other neonicotinoids causing similar effects (Annoscia et al., 2020; Brandt et al., 2016). Our finding of an effect on gene expression of clothianidin at 5 ppb is in keeping with this. The effect we observed could have been an indirect effect, for example from clothianidin inducing detoxification pathways, but it is in keeping with the direct effect of the neonicotinoid on *ACh* signalling in haemocytes that would be predicted by the haemocytes having *nAChR* receptors. The fact that clothianidin increased expression of *ChAT* could suggest that it will produce an increase in the production of antimicrobial peptides and therefore resistance to disease (Giordani et al., 2023; Hanson & Lemaitre, 2023). Low levels of stress, including from pesticides, can result in increased gene expression and stimulatory effects on a diversity of biological functions (Rix & Cutler, 2022). However, this would be contrary to the immunosuppressive effects of clothianidin and other neonicotinoids that have been abundantly demonstrated (Annoscia et al., 2020; Brandt et al., 2016; Di Prisco et al., 2013; López et al., 2017; Malladi et al., 2023; Orčić et al., 2022). An alternative explanation is that the clothianidin induces overstimulation of the *nAChR* receptors or off-target synthesis of choline acetyltransferase and acetylcholine that negatively impacts cell function and homeostasis. Neonicotinoid insecticides are designed to target *nAChR* receptors with high affinity (Elbert et al., 2008; Matsuda et al., 2001), causing lethal effects through receptor overstimulation (Tomizawa & Casida, 2003; Tomizawa, Lee & Casida, 2000). In molluscs, the blocking of haemocyte-based *mAChR* before pathogen challenge promotes the expression of *Tumor Necrosis Factor* (TNF), which in turn results in elevated haemocyte apoptosis (Liu et al., 2016a, 2016b). If a similar, *nAChR*-based, regulatory connection is present in the haemocytes of pollinators, *nAChR* blockage by neonicotinoids could directly explain their detrimental effects on haemocytes and by extension the immunosuppressive effects observed in honeybees (Brandt et al., 2017; Di Prisco et al., 2013; Malladi et al., 2023). In addition to confirming the presence of functional receptors and ACh communication in the non-neural tissues of bees, future work should investigate the effects of neonicotinoid exposure on expression of the *nAChR* subunits and other components of the acetylcholine signalling machinery in haemocytes, and the downstream impacts of these effects. Our results suggest that haemocytes may use different receptor subunits than the brain, so determining the relative sensitivity of haemocytes to neonicotinoids compared with other tissues also warrants further investigation.

While the direct effects of neonicotinoids on neuronally-associated traits such as behaviour, memory and navigation are intuitive (Blacquière et al., 2012; Fischer et al., 2014; Jin et al., 2015), the effects on other traits such as immunity or reproduction have not previously been adequately explained (Straub et al., 2016; Whitehorn et al., 2012; Williams et al., 2015). The finding that non-neural tissues including haemocytes can potentially express *nAChR* could explain these counterintuitive effects by providing a mechanism for direct interaction with these tissues (Pamminger et al., 2018). These systemic pesticides migrate into both pollen and nectar, so pollinators are exposed to them when visiting treated crops or contaminated wildflowers (Botias et al., 2015; Goulson et al., 2015). Once ingested, the pesticide is absorbed *via* the gut and passes through the haemolymph on the way to its designated target sites in the central nervous system (Tomizawa & Casida, 2003). In the haemolymph, neonicotinoids inevitably come into contact with haemocytes, with potentially disruptive effects for haemocyte function if haemocytes are sensitive to neonicotinoids. In addition, the differences between tissues in the relative expression patterns of *nAChR* subunits could help to explain the pronounced differences in susceptibility to neonicotinoids between different developmental stages, species and experiments (Grewal, Power & Shetlar, 2001; Moffat et al., 2016; Tomizawa & Casida, 2003; Whitehorn et al., 2012). Since the subunit composition determines the binding properties and consequently toxicity of neonicotinoids, and such composition varies between species, tissues, life stages and time, this variation could explain the observed differences in toxicity by orders of magnitude (Govind, Vezina & Green, 2009; Moffat et al., 2016; Tomizawa & Casida, 2003, 2009; Whitehorn et al., 2012; Xu et al., 2017).

## Conclusions

In summary, our results support a mechanistically informed framework to understand the numerous unexplained side effects associated with sub-lethal neurotoxic pesticides exposure in pollinators. Such an analysis framework is urgently needed in order to identify and ultimately limit the numerous side effects of neurotoxic pesticides.

## Supplemental Information

10.7717/peerj.17978/supp-1 Supplemental Information 1 Supplemental Tables.

10.7717/peerj.17978/supp-2 Supplemental Information 2 Raw data.

## Additional Information and Declarations

### Data Availability

The following information was supplied regarding data availability: The raw data is available in the Supplemental File.
