# Supplementary material for: Potential acetylcholine-based communication in honeybee haemocytes and its modulation by a neonicotinoid insecticide
Source: PeerJ. 2024 Sep 13;12:e17978. doi: 10.7717/peerj.17978 (PMC11404474; doi:10.7717/peerj.17978)
Supplement: Supplemental Information 1 [file peerj-12-17978-s001.pdf]

Table S1.: Results of the PERMANOVA pairwise comparisons of nAChR subunit expression based on Chad distance and the overall results when using euclidian distance as comparison. Presented are Pseudo-F, t and P values

	Haemocyte	Fatbody	Midgut
Brain	t = 2.302; P = 0.0011	t = 3.842; P = 0.0013	t = 3.586; P < 0.001
Haemocyte		t = 2.404; P = 0.0014	t = 1.922; P = 0.006
Fatbody			t = 3.41; P = < 0.001

Euclidian distance

Overall effect	Pseudo F = 6,2202	P < 0.001	
Pairwise comparison			
	Haemocyte	Fatbody	Midgut
Brain	t = 2.33; P = 0.0013	t = 2.972; P < 0.001	t = 2.82; P = 0.0011
Haemocyte		t = 2.175; P < 0.001	t = 1.39; P = 0.044
Fatbody			t = 3.27; P = < 0.001

Table S2.: Detailed results of the SIMPER (similarity percentages) analysis. Presented are the contributions of the nAChR subunits contributing more then 10% to within- and between group (tissue) variation

	Within group variation (avg. squared distance)	nNAChR/ contribution%	nNAChR/ contribution%	nNAChR/ contribution%	nNAChR/ contribution%	nNAChR/ contribution%
Fatbody	3,65	a9/48.85	b2/40.23%			
Haemocyte	8,86	a8/27.14%	a1/23.85%	a6/19.17%	a2/14.36%	
Midgut	0,90	a1/68%	a6/22.45%			
Brain	17,15	b1/26.83%	a3/15.71%	a6/13.9%	a4/12.84%	a5/11.47%
	**Between group variation (avg. squared distance)**	**nNAChR/ contribution%**	**nNAChR/ contribution%**	**nNAChR/ contribution%**	**nNAChR/ contribution%**	**nNAChR/ contribution%**
Fatbody vs. Haemocyte	19,05	a9/21.66%	b2/20.67%	a8/15.68%	a1/14.74%	
Fatbody vs. Midgut	10,86	a9/41.03%	b2/40.1%	a1/12.7%		
Fatbody vs. Brain	44,24	a7/14.34%	b1/12.74%	b2/11.41%	a5/11.4%	a9/10.62%
Haemocyte vs. Midgut	10,86	a9/41.03%	b2/40.1%	a1/12.7%		
Haemocyte vs. Brain	42,56	a5/15.38%	a4/15.06%	a7/14.88%	a3/13.91%	b1/11.67%
Midgut vs. Brain	34,7	a7/18.28%	a5/16.35%	b1/16.2%	a2/12.07%	a3/11.95%

Table S3: Details on qPCR primer design including primer target, sequence, annealing temperature (C), genebank reference if available and journal article cited

primer name	gene	5'->3' sequence	TM	genebank ref.	literature
A.mellifera_nAChRa1_f	nAChR subunit a1	CGACCTGCTGTCCAACTACA	59,9	gi|148277573	Genome Res. 16 (11), 1422-1430 (2006)
A.mellifera_nAChRa1_r	nAChR subunit a1	ACTCTTGTTCCACCCAAACG	60,01	gi|148277573	Genome Res. 16 (11), 1422-1430 (2006)
A.mellifera_nAChRa2_f	nAChR subunit a2	CGATGACCAAAGCCATCCTG	58,98	gi|402745439	Eur. J. Neurosci. 36 (10), 3438-3450 (2012)
A.mellifera_nAChRa2_r	nAChR subunit a2	TACGTCCAGGAACCGAACTT	58,67	gi|402745439	Eur. J. Neurosci. 36 (10), 3438-3450 (2012)
A.mellifera_nAChRa3_f	nAChR subunit a3	CAAGACTACTGGTGATGCGC	59	gi|118601177	Genome Res. 16 (11), 1422-1430 (2006)
A.mellifera_nAChRa3_r	nAChR subunit a3	TTCGAGACCATTGCACGTTC	58,86	gi|118601177	Genome Res. 16 (11), 1422-1430 (2006)
A.mellifera_nAChRa4_f	nAChR subunit a4	ATCATTCCACCTACGTCGCT	58,89	GI:148277658	Genome Res. 16 (11), 1422-1430 (2006)
A.mellifera_nAChRa4_r	nAChR subunit a4	CCCAAGGTGCCATTTTGTGA	58,95	GI:148277658	Genome Res. 16 (11), 1422-1430 (2006)
A.mellifera_nAChRa5_f	nAChR subunit a5	AGTTCTTCCCCTTCGACGAG	59,11	gi|748944262	NA
A.mellifera_nAChRa5_r	nAChR subunit a5	CTCGCGGAGAAATTGACCAG	59	gi|748944262	NA
A.mellifera_nAChRa6_f	nAChR subunit a6	GTGTTCCTGAATCTCGTCGC	59,01	gi|121583857	BMC Evol. Biol. 7, 98 (2007)
A.mellifera_nAChRa6_r	nAChR subunit a6	CGGGCGTTCTGTGATGAAAA	59,13	gi|121583857	BMC Evol. Biol. 7, 98 (2007)
A.mellifera_nAChRa7_f	nAChR subunit a7	CCACTGGCACACTCCTCTTA	59,02	gi|58585185	Eur. J. Neurosci. 36 (10), 3438-3450 (2012)
A.mellifera_nAChRa7_r	nAChR subunit a7	CATCATGAGCTCGTTTCGGG	59,07	gi|58585185	Eur. J. Neurosci. 36 (10), 3438-3450 (2012)
A.mellifera_nAChRa8_f	nAChR subunit a8	GGTCATTGCCATCTCAACCC	58,89	gi|58585099	Eur. J. Neurosci. 36 (10), 3438-3450 (2012)

A.mellifera_nAChRa8_r	nAChR subunit a8	TGTGTCAGGATTTGCTTCGA	57,46	gi|58585099	Eur. J. Neurosci. 36 (10), 3438-3450 (2012)
A.mellifera_nAChRa9_f	nAChR subunit a9	TGACTCTCATGTGGACCGAC	60	Gi|402745352	Genome Res. 16 (11), 1422-1430 (2006)
A.mellifera_nAChRa9_r	nAChR subunit a9	TTGATCGAACGTCATGTCGC	60	gi|402745352	Genome Res. 16 (11), 1422-1430 (2006)
A.mellifera_nAChRb1_f	nAChR subunit b1	GGAAGGTTCATCACACGACG	58,93	gi|118601181	J. Neurophysiol. 106 (4), 1604-1613 (2011)
A.mellifera_nAChRb1_r	nAChR subunit b1	CTCGGCAATGAACTCGACAG	59,01	gi|118601181	J. Neurophysiol. 106 (4), 1604-1613 (2011)
A.mellifera_nAChRb2_f	nAChR subunit b2	CACCGTCGATTTCCACGTTA	58,3	gi|148277645	Genome Res. 16 (11), 1422-1430 (2006)
A.mellifera_nAChRb2_r	nAChR subunit b2	GATACCGACGCTTGTTACGC	59,43	gi|148277645	Genome Res. 16 (11), 1422-1430 (2006)
A.mellifera_rp49_f	rp49	CGTCATATGTTGCCAACTGG	60	NA	*Apidologie* 39, no. 3 (2008): 372-385.
A.mellifera_rp49_r	rp49	TTGAGCACGTTCAACAATGG	60	NA	*Apidologie* 39, no. 3 (2008): 372-385.
AM_choli_O_tran_f	choline acetyltransferase	GTCTGTGCGTGGTGTGTATC	58,93	gi|1032026178	BMC Genomics 15, 86 (2014)
AM_choli_O_tran_r	choline acetyltransferase	GAACCTCCGCCATGAAGAAC	58,91	gi|1032026178	BMC Genomics 15, 86 (2014)
